# Superior growth performance in broiler chicks fed chelated compared to inorganic zinc in presence of elevated dietary copper

**DOI:** 10.1186/s40104-016-0072-1

**Published:** 2016-02-29

**Authors:** Junmei Zhao, Robert B. Shirley, Julia J. Dibner, Karen J. Wedekind, Frances Yan, Paula Fisher, Thomas R. Hampton, Joseph L. Evans, Mercedes Vazquez-Añon

**Affiliations:** Research and Development, Novus International, 20 Research Park Drive, Saint Charles, MO 63304 USA; Current affiliation: Poultry Technical Services, Adisseo USA, Alpharetta, GA 30022 USA

**Keywords:** Antagonism, Broilers, Chelates, Growth, Minerals, Organic, Performance

## Abstract

**Background:**

The goal of this study was to compare the antagonism of elevated dietary Cu (250 mg/kg) from CuSO_4_ on three different Zn sources (ZnSO_4_ · H_2_O; [Zn bis(−2-hydroxy-4-(methylthio)butanoic acid)], Zn(HMTBa)_2_, a chelated Zn methionine hydroxy analogue; and Zn-Methionine), as measured using multiple indices of animal performance in ROSS 308 broilers.

**Methods:**

Three experiments were conducted in broiler chicks fed a semi-purified diet. All birds were fed a Zn-deficient diet (8.5 mg/kg diet) for 1 wk, and then provided with the experimental diets for 2 wks.

**Results:**

Experiment 1 was a 2 × 2 factorial design with two levels of Cu (8 vs. 250 mg/kg diet from CuSO_4_) and two Zn sources at 30 mg/kg [ZnSO_4_ · H_2_O vs. Zn(HMTBa)_2_]. Elevated Cu impaired growth performance only in birds fed ZnSO_4_. Compared to ZnSO_4_ · H_2_O, Zn(HMTBa)_2_ improved feed intake (12 %; *P* < 0.001) and weight gain (12 %, *P* < 0.001) and the benefits were more pronounced in the presence of 250 mg/kg diet Cu. Experiment 2 was a dose titration of ZnSO_4_ · H_2_O and Zn(HMTBa)_2_ at 30, 45, 60, and 75 mg/kg diet in the presence of 250 mg/kg CuSO_4_. Feed:gain was decreased and tibia Zn was increased with increasing Zn levels from 30 to 75 mg/kg. Birds fed Zn(HMTBa)_2_ consumed more food and gained more weight compared to birds fed ZnSO_4_, especially at lower supplementation levels (30 and 45 mg/kg; interaction *P* < 0,05). Experiment 3 compared two organic Zn sources (Zn(HMTBa)_2_ vs. Zn-Methionine) at 30 mg/kg with or without 250 mg/kg CuSO_4_. No interactions were observed between Zn sources and Cu levels on performance or tissue mineral concentrations. High dietary Cu decreased weight gain (*P* < 0.01). Tibia Cu and liver Cu were significantly increased with 250 mg/kg dietary Cu supplementation (*P* < 0.01). No difference was observed between the two Zn sources.

**Conclusions:**

Dietary 250 mg/kg Cu significantly impaired feed intake and weight gain in birds fed ZnSO_4_ · H_2_O_,_ but had less impact in birds fed Zn(HMTBa)_2_. No difference was observed between the two organic zinc sources. These results are consistent with the hypothesis that chelated organic Zn is better utilized than inorganic zinc in the presence of elevated Cu.

## Background

Elevated dietary CuSO_4_ (125–250 mg/kg diet) is often used in broiler and pig diets as a growth promoter and anti-bacterial agent, possibly by modulating the microbial population within the gastrointestinal tract [1, 2]. However, high dietary Cu can reduce the bioavailability of other important nutrients by forming insoluble complexes or by competing for absorption sites. The reciprocal antagonism between Zn and Cu is a prime example of competitive biological interactions between metals due to their similar chemical and physical properties [3, 4]. Excessive Zn supply has been shown to inhibit intestinal absorption, hepatic accumulation, and placental transfer of Cu, as well as to induce clinical and biochemical signs of Cu deficiency [5, 6]. The intestinal absorption of ^65^Zn was decreased by approximately 20 % when the dietary Cu intake was increased from 3 to 24 mg/kg diet. The mechanism(s) of Zn and Cu interaction is not well understood, and the data are controversial to interpret. They compete for uptake sites in the mucosa, and are regulated by the same metallothionein protein. In addition, they might impact each other’s solubility and binding behavior in gastrointestinal tract. Pang and Applegate indicated that high Cu supplementation (250 mg/kg in diet) increased the percentage of Zn associated with large complexes (>100,000 MW), and decreased the percentage of Zn associated with small complexes (<5,000 MW; *P* < 0.05), thereby suggesting an antagonism between Cu and Zn [7]. The association of Zn with smaller complexes might enhance the potential for absorption due to the larger surface area.

Most previous studies of dietary Zn-Cu antagonism have evaluated inorganic Zn and inorganic Cu. The availability of chelated organic trace minerals (OTM) in the marketplace now offers a potential solution to the inherent limitations associated with the widespread commercial practice of employing inorganic dietary CuSO_4_, by reducing and possibly avoiding the antagonism between Cu and Zn. Results from recent studies indicate that OTM might be more available for absorption, likely due to reduced incidence of antagonistic reactions with other dietary constituents in the gastrointestinal tract [8–10]. The relative bioavailability of a chelated Zn methionine hydroxy analogue [Zn bis(−2-hydroxy-4-(methylthio)butanoic acid; Zn(HMTBa)_2_] (compared to ZnSO_4_) in broilers was reported to be 161 % based on tibia Zn, and 248 % based on metallothionein mRNA expression [11]. When dietary Ca and P were increased, a common practice in diets of layers and pets, the relative bioavailability of chelated Zn increased to 441 % (based on μg total tibia Zn). The authors suggested that the difference in relative bioavailability might be attributed to the fact that chelated Zn possesses stronger chemical (coordinate-covalent) bonds relative to ZnSO_4_ and, thus, is more resistant to antagonisms in gastrointestinal tract. However, published literature on relative bioavailability of different sources of Zn is mixed. The inconsistencies could be due to differences in the chemical characteristics (and thus bioavailability) between different organic Zn forms. Adding to the complexity of comparing results from different studies using various forms of Zn is the inconsistency of terminology (organic, chelates, complex, etc.) along with the difficulty of characterizing chemical structures.

The primary objectives of the experiments reported here were to investigate the potential antagonism imposed by high dietary CuSO_4_ on Zn bioavailability in broilers, and to evaluate whether different Zn sources impact the Cu-mediated antagonism. We hypothesized that chelated Zn is more resistant to high Cu antagonism due to their stable structure and resistance to interactions with other nutrients in the gastrointestinal tract. Three Zn sources were used in the experiments: ZnSO_4_ · H_2_O, Zn(HMTBa)_2_, and Zn-methionine (Zn-MET). Zn(HMTBa)_2_ is a chelated Zn methionine hydroxy analogue [Zn bis(−2-hydroxy-4-(methylthio)butanoic acid)] at 2:1 ratio. To the best of our knowledge, this is the first report of the evaluation of the impact of high Cu on the utilization of different Zn sources in broilers.

## Methods

The animal protocols for all experiments were approved by the Novus International Institutional Animal Care and Use Committee, and complied with all federal and state statutes ensuring the humane and ethical treatment of experimental animals. Diet formulation and *in vivo* activities were performed at the Novus International Research Farm. ZnSO_4_ · H_2_O was purchased from Sigma-Aldrich (Saint Louis, MO USA). Zn(HMTBa)_2_ (MINTREX® Zn, Novus International Inc, Saint Charles, MO USA) is a chelate of one Zn with two molecules of 2-hydroxy-4-(methylthio)butanoic acid (HMTBa), and was obtained from Novus International. Zinc methionine complex (Zn-MET) is the product resulting from complexing a soluble metal salt with methionine, and was purchased from Zinpro (Eden Prairie, MN USA) [12].

Three consecutive experiments were conducted. Experiment 1 was designed to determine whether 250 mg/kg dietary Cu from CuSO_4_ antagonizes two different Zn sources [ZnSO_4_ · H_2_O vs. Zn(HMTBa)_2_]. ZnSO_4_ · H_2_O and Zn(HMTBa)_2_ were added at 21.5 mg/kg to achieve 30 mg/kg total Zn in the finished feed (the other 8.5 mg/kg came from feed ingredients). Similar calculations were used in Experiments 2 and 3, such that different Zn sources were added on top of the basal to achieve the targeted Zn concentration in the final feed. A total of 288 ROSS 308 male chicks were randomly divided into four experimental treatments with six replicate pens per treatment and 12 birds per pen. The trial was a 2 × 2 factorial design with two levels of Cu (8 vs. 250 mg/kg diet from CuSO_4_) and two Zn sources at 30 mg/kg [ZnSO_4_ · H_2_O vs Zn(HMTBa)_2_]. Based on Experiment 1, Experiment 2 was designed to compare ZnSO_4_ · H_2_O and Zn(HMTBa)_2_ in a dose titration in the presence of 250 mg/kg diet CuSO_4_. A total of 576 ROSS 308 male chicks were allotted to eight experimental treatments with six replicate pens per treatment and 12 birds per pen. The dietary treatments included either ZnSO_4_ · H_2_O or Zn(HMTBa)_2_ added to achieve the following dietary levels: 30, 45, 60, and 75 mg/kg Zn. All diets contained 250 mg/kg diet Cu from CuSO_4_. Based on the results of the first two experiments, Experiment 3 was designed to compare two different organic Zn sources, Zn(HMTBa)_2_ and Zn-MET, on performance in the presence of 8 vs. 250 mg/kg CuSO_4_ dietary supplementation. A total of 480 day-old ROSS 308 male broilers were allotted to four treatments, with 8 replicate pens per treatment and 15 birds per pen. The trial was a 2 × 2 factorial design with two levels of Cu (8 and 250 mg/kg diet as CuSO_4_) and two Zn sources [Zn(HMTBa)_2_ vs. Zn-MET] at 30 mg/kg diet.

Birds were housed in stainless steel pens in a thermostatically controlled, electrically heated environment. The dimension of each battery pen was 51 × 69 × 35 cm (width, length, and height, respectively). Each pen was provided with water and an individual feeder. All birds were allowed to consume mash feed and water ad libitum. Room temperature was kept at 32 °C for the first two days, then reduced until a temperature of 23 °C was reached on d 17 of age. The light–dark cycle was as follows: on d 0 through d 7, there were 23 h of light and 1 h of darkness (lights off at 1200 h and on at 0100 h). On d 8 through d 22, there were 20 h of light and 4 h of darkness (lights off at 1200 h and on at 0400 h). All birds were fed a common semi-purified Zn-deficient diet (8.5 mg/kg diet) for the first week to reduce Zn stores and then were fed the experimental diets for about 2 wks (basal diet formula, see Table 1). A common semi-purified basal diet was used and formulated to meet National Research Council (1994) dietary recommendations for all nutrients except for Zn [13]. Each dietary treatment was then made by adding different Zn sources and Cu to the common basal. Methionine activity from Zn(HMTBa)_2_ (80 % methionine activity) [14], and Zn-MET (20 % methionine activity) was accounted for, and all diets were adjusted to have iso-methionine level.

**Table 1:** Basal diet formula and nutrient profile (Experiments 1 through 3)

Ingredients	g/kg	Nutrients, g/kg	
Dextrose	317.5	Metabolize energy, MJ/kg	13.0
Corn starch	158.4	Crude protein	222.6
Soy concentrate, 84 %	152.8	Calcium	9.0
Cellulose	124.3	Total phosphorus	6.2
Soy protein concentrate	110.0	Available phosphorus	4.5
Soybean oil	50	Available amino acid	
Milo (grain sorghum)^a^	45	Lysine	11.5
Calcium phosphate	14.5	Methionine	6.8
MHA^b^	5.5	Methionine + cystine	8.3
Potassium phosphate	4.8		
Calcium carbonate	3.3		
Mineral premix^c^	2.5		
Choline, 70 %	2.3		
Potassium chloride	1.7		
Sodium chloride	1.6		
Magnesium sulfate heptahydrate	1.4		
Threonine	1.1		
Vitamin premix^d^	0.6		
L-lysine, 78 %	0.6		
Sodium bicarbonate	0.3		
Tryptophan	0.2		
Santoquin-Mix 6	0.1		
Silica^e^	1.5		

^a^Milo, a draught-tolerant crop also known as grain sorghum, is a food product for humans and livestock feed grain. In the US, the feed grain is mainly for poultry and cattle

^b^MHA is the calcium salt of 2-hydroxy-4 methylthio butanoic acid (Novus International), providing 84 % methionine activity. MHA inclusion rate was adjusted to reflect methionine activity provided from Zn(HMTBa)_2_ (80 % methionine activity) and Zn-MET (20 % methionine activity) to maintain an equal methionine level across all treatments

^c^The mineral premix provided in the final diet (mg/kg): 60 Mn (MnO); 80 Fe (FeSO_4_ · 7H_2_O); 8 Cu (CuSO_4_ · 5H_2_O); 0.35 I (ED Iodide); 0.15 Se (NaSeO_3_). Analyzed final basal diet Zn was 8.5 mg/kg

^d^Vitamin premix provided in the final diet (mg/kg): retinylacetate 10; cholecalciferol 6.25; dl-alpha tocopheryl acetate 90; menadione sodium bisulphate 7; calcium pantothenate 10; cyanocobalamin, 1.4; riboflavin 6.5; niacin 37; biotin 0.15, thiamin monoitrate 2.25; pyridoxine 4.25; and folic acid 0.9

^e^Silica was removed from the diet as different Zn sources were added

Body weight and feed intake were recorded at beginning and the end of each experiment. On d 21 (d 19 for Experiment 3), one chick per cage (selected based on body weight approximating the pen mean) was chosen, euthanized with CO_2_, and the left tibia (without cartilage cap) and liver (Experiment 3)were collected for Zn and Cu (Experiment 3) analyses. Following the careful removal of muscle and connective tissue, the whole tibia was ashed. Tibia Zn and Cu were measured at the Novus International Analytical Services Laboratories (Saint Charles, MO USA) using Inductively Coupled Plasma Optical Emission Spectrometry (ICP-OES, Perkin Elmer, Shelton, CT USA) following an internally validated method based on AOAC 985.01 [15]. Tibia Zn and Cu level were reported on a dry ash weight basis, and liver Zn and Cu levels reported on a dry matter basis.

### Statistical analyses

In Experiments 1 and 3, data were analyzed with a 2-way ANOVA using the General Linear Models (GLM) procedure of SAS (version 9.1; Cary, NC USA). The model included the main effects of Zn sources, Cu levels (with and without high CuSO_4_), and their interaction. In Experiment 2, data were analyzed with a 2-way ANOVA using the GLM procedure. The model included the main effect of two Zn sources [Zn(HMTBa)_2_ vs. ZnSO_4_ · H_2_O], and four different supplementation levels, and their 2-way interaction. Means were separated by Fisher’s protected least significant difference method when the F test was significant. Mortality data were transformed to square root of mortality + 1 before analysis. Data are presented in natural numbers. Pen served as the experimental unit. Effects were considered significant at 95 % probability (*P* ≤ 0.05).

## Results

### Experiment 1

As shown in Table 2, significant two-way interactions of Cu level and Zn source were observed on body weight (*P* < 0.05) and weight gain (*P* < 0.05). Compared to 8 mg/kg CuSO_4_, weight gain was decreased 14 % with 250 mg/kg Cu supplementation in the ZnSO_4_ · H_2_O groups (382 vs. 328 g), but not in the Zn(HMTBa)_2_ groups (404 vs. 390 g). Similar results were observed on final body weight, where 250 mg/kg dietary Cu reduced final body weight in ZnSO_4_ · H_2_O (465 vs. 407 g) but not in the Zn(HMTBa)_2_ groups (481 vs. 467 g). No interaction was observed on feed intake (*P* = 0.34, Table 2). For main effect, dietary 250 mg/kg CuSO_4_ decreased feed intake 10 % compared to the 8 mg/kg CuSO_4_ control groups regardless of Zn source (*P* < 0.001, Table 2). Compared to ZnSO_4_ · H_2_O, birds fed Zn(HMTBa)_2_ consumed 12 % more feed (*P* < 0.001). Feed conversion, mortality, and tibia Zn were not affected by dietary treatments (*P* > 0.50). In summary, dietary Cu from CuSO_4_ impaired feed intake across Zn sources, but decreased weight gain only in birds fed ZnSO_4_ · H_2_O. Compared to ZnSO_4_ · H_2_O, Zn(HMTBa)_2_ improved weight gain and feed intake, and the benefits were more profound with 250 mg/kg dietary Cu supplementation.

**Table 2:** Effects of Zn sources and CuSO_4_ on growth performance and tibia Zn in broilers (Experiment 1)^1^

Cu levels, mg/kg	Zn Source, 30 mg/kg	D7 BW, g/bird	D21 BW, g/bird	Gain, g/bird	Feed Intake, g/bird	Feed:gain	Mortality, %	Ash based tibia Zn, mg/kg^2^
8	ZnSO_4_	83	465^a^	382^b^	523	1.372	2.78	148
250	ZnSO_4_	78	407^b^	328^c^	456	1.389	1.39	158
8	Zn(HMTBa)_2_	77	481^a^	404^a^	569	1.407	5.55	170
250	Zn(HMTBa)_2_	78	467^a^	390^ab^	528	1.355	2.78	150
SEM		2	8	7	13	0.027	2.26	14
Main effect means								
Cu level								
	8	79	473	393	546^a^	1.390	4.167	159
	250	78	437	359	492^b^	1.372	2.083	154
Zn source								
	ZnSO_4_	80	436	355	489^b^	1.381	2.083	153
	Zn(HMTBa)_2_	77	474	397	548^a^	1.381	4.165	160
	2x2 factorial arrangement, *P* value							
Zn sources		--	<0.001	<0.001	<0.001	0.980	0.368	0.597
Cu level		--	<0.001	<0.001	<0.001	0.515	0.367	0.722
Zn Source * Cu level		--	0.012	0.011	0.334	0.208	0.762	0.298

^1^A total of 288 ROSS 308 male birds were used, with six cages per treatment and 12 birds per pen

^2^Tibias were collected from one bird per pen, total of six birds per treatment

^a,b,c^Means within a column with no common superscripts differ (*P* < 0.05)

### Experiment 2

As shown in Table 3, significant two-way interactions of Zn source and level were observed on body weight (*P* = 0.02), weight gain (*P* = 0.005), and feed intake (*P* < 0.001, Table 3). The superiority of Zn(HMTBa)_2_ over ZnSO_4_ · H_2_O was more pronounced at lower levels of Zn supplementation. Birds achieved maximal performance at 45 mg/kg Zn supplementation from Zn(HMTBa)_2_, and no further improvement was observed at levels above that (Table 3). In contrast, performance was linearly improved in birds fed Zn from ZnSO_4_ · H_2_O from 30 to 75 mg/kg, with the best performance at the highest level. Feed:gain was decreased and tibia Zn was increased with increased Zn level regardless of the source (main effect, *P* < 0.05). No differences were observed on tibia Zn or mortality between Zn sources (*P* > 0.05). In summary, birds fed Zn(HMTBa)_2_ ate more and gained more compared to birds fed ZnSO_4_ · H_2_O, especially at lower levels of Zn supplementation. Less Zn(HMTBa)_2_ was needed to achieve similar performance compared to ZnSO_4_ · H_2_O. Feed efficiency and tibia Zn increased with increased dietary Zn in the range of 30 to 75 mg/kg.

**Table 3:** Effects of Zn sources with high dietary CuSO_4_ on performance and tibia Zn in broilers (Experiment 2)^1^

Zn level, mg/kg	Zn Source	CuSO_4_, mg/kg	D7 BW, g/bird	D21 BW, g/bird	Gain, g/bird	Feed Intake, g/bird	Feed:gain	Mortality, %	Ash based tibia Zn, mg/kg^2^
30	ZnSO_4_	250	78	406^d^	328^d^	455^c^	1.388	1.54	158
45	ZnSO_4_	250	78	488^bc^	411^bc^	550^a^	1.341	1.39	219
60	ZnSO_4_	250	76	501^ab^	425^ab^	557^a^	1.315	0.00	256
75	ZnSO_4_	250	78	515^a^	438^a^	571^a^	1.307	0.00	263
30	Zn(HMTBa)_2_	250	78	468^c^	390^c^	527^b^	1.355	2.78	150
45	Zn(HMTBa)_2_	250	80	519^a^	440^a^	571^a^	1.302	0.00	228
60	Zn(HMTBa)_2_	250	80	514^ab^	434^a^	566^a^	1.304	1.24	271
75	Zn(HMTBa)_2_	250	77	519^a^	441^a^	572^a^	1.300	1.24	267
SEM			2	9	8	8	0.020	1.16	12
Main effect means									
Zn source									
	ZnSO_4_		78	475	396	531	1.345	1.111	208
	Zn(HMTBa)_2_		78	500	422	561	1.333	2.220	217
Zn level									
	30		78	455	376	519	1.381^a^	3.125	157^c^
	45		79	504	425	561	1.321^b^	0.694	224^b^
	60		78	507	429	561	1.309^b^	0.694	263^a^
	75		77	517	439	572	1.303^b^	0.694	265^a^
2 × 4 factorial arrangement, *P* value									
Zn level			--	<0.001	<0.001	<0.001	0.004	0.549	<0.001
Zn Source			--	<0.001	<0.001	<0.001	0.102	0.403	0.569
Zn source * level			--	0.021	0.005	<0.001	0.827	0.549	0.833

^1^A total of 576 ROSS 308 male chicks were allotted to eight treatments with six replicate cages per treatment and 12 birds per cage.

^2^Tibias were collected from one bird per pen, total six birds per treatment

^a-d^Means within a column with no common superscripts differ (*P* < 0.05)

### Experiment 3

No interactions between Cu levels and Zn sources were observed on performance (*P* > 0.25; Table 4). No differences were observed on feed intake, feed efficiency, and mortality among treatments (*P* > 0.25, Table 4). Similar to experiment 1, birds fed 250 mg/kg CuSO_4_ gained less weight and were lighter compared to birds fed 8 mg/kg CuSO_4_ (main Cu effect, *P* < 0.05). Tibia Zn and liver Zn were not different among treatments (*P* > 0.50, Table 5). Dietary Cu supplementation significantly increased bone and hepatic Cu level, regardless of Zn sources (*P* < 0.001, Table 5). Liver Cu was increased greater than 10 fold with 250 mg/kg Cu supplementation. In summary, 250 mg/kg Cu decreased weight gain and significantly increased liver and tibia Cu concentration. No difference was observed between the two Zn sources.

**Table 4:** Effects of organic Zn sources and high dietary CuSO_4_ on growth performance in broilers (Experiment 3)^1^

CuSO_4_, mg/kg	Zn source, 30 mg/kg	D8 BW, g/bird	D19 BW, g/bird	Gain, g/bird	Feed Intake, g/bird	Feed:gain	Mortality, %
8	Zn(HMTBa)_2_	83	375	336	457	1.362	1.67
250	Zn(HMTBa)_2_	82	367	328	438	1.336	0.83
8	Zn-MET	81	374	335	457	1.364	1.67
250	Zn-MET	80	356	317	447	1.408	2.50
SEM		1	4	4	12	0.030	1.39
Main effect means							
Cu level							
	8	82	375^a^	335^a^	457	1.363	1.77
	250	82	361^b^	332^b^	442	1.372	1.77
Zn source							
	Zn(HMTBa)_2_	82	371	332	447	1.349	1.25
	Zn-MET	81	365	326	452	1.385	2.18
2 × 2 factorial arrangement, *P* value							
Zn source		--	0.187	0.198	0.703	0.217	0.553
Cu level		--	0.004	0.005	0.227	0.759	1.000
Zn Source* Cu level		--	0.292	0.271	0.696	0.245	0.554

^1^A total of 288 ROSS 308 male birds were used, with six cages per treatment, and 12 birds per pen

^a,b^Means within a column with no common superscripts differ (*P* < 0.05)

**Table 5:** Effects of Zn sources and dietary CuSO_4_ on tissue mineral content (Experiment 3)^1^

Cu levels, mg/kg	Zn sources, 30 mg/kg	Tibia Zn (ash based), mg/kg	Tibia Cu (ash based), mg/kg	Liver Zn (dry wt. based), mg/kg	Liver Cu (dry wt. based), mg/kg
8	Zn(HMTBa)_2_	143	6.5	104	13.5
250	Zn(HMTBa)_2_	154	11.8	69	169.7
8	Zn-MET	140	6.0	93	13.3
250	Zn-MET	147	10.2	57	128.2
SEM		8	0.8	24	19.8
Main effect means					
Cu level					
	8	141	6.23^b^	98	13.4^b^
	250	150	11.0^a^	63	150^a^
Zn source					
	Zn(HMTBa)_2_	148	9.14	86	91.6
	ZnSO_4_	143	8.11	75	70.8
2 × 2 factorial arrangement, *P* value					
Zn source		0.543	0.229	0.653	0.296
Cu level		0.231	<0.001	0.168	<0.001
Zn Source* Cu level		0.780	0.513	0.981	0.301

^1^ One bird per pen was killed for tissue collection, total of six birds per treatment

^a,b^Means within a column with no common superscripts differ (*P* < 0.05)

## Discussion

The data reported here are consistent with the hypothesis that chelated organic Zn forms are more efficient, compared to an inorganic zinc salt, in their abilities to overcome the antagonism mediated by elevated dietary Cu. Collectively, the data from our study indicate that a chelated form of Zn [Zn(HMTBa)_2_] outperforms the inorganic ZnSO_4_ · H_2_O, in the context of high dietary Cu-mediated antagonism in broiler chicks. Similarly, Richards et al. recently reported that Zn(HMTBa)_2_ is more bioavailable than ZnSO_4,_ and the difference is greater under conditions of elevated dietary Ca and P supplementation [11].

To the best of our knowledge, this is the first report demonstrating that high dietary Cu in broilers antagonizes, to differing degrees, different dietary sources of Zn. We used a soy isolate/soy concentrate diet to assess the relative performance differences between zinc sources in chicks. The use of such a diet is an accepted model to assess relative bioavailability or performance differences between Zn sources [8]. The presence of phytate and fiber (similar to corn-soybean meal diets) makes this semi-purified diet more relevant to commercial diets compared to a crystalline amino acid, casein-dextrose, or egg white diet which are devoid of phytate.

The reciprocal antagonisms between dietary Zn and Cu are well known. For example, high dietary Cu (204 mg Cu/kg for 10 d) retarded growth performance in turkey poults [16]. Hall et al. reported a 20 % decrease in ^65^Zn absorption when dietary Cu was raised from 3 to 24 mg/kg in rats [5]. The mechanism(s) by which Cu antagonizes Zn is not well understood, although it has been postulated that these antagonisms are due to their similar chemical and physical properties [3]. It seems unlikely that Cu-Zn interaction occurs at a systemic level, because a) the absorption of these two metals occurs via different transporters, b) their functions and metabolism are quite different, and c) they do not share common storage proteins except metallothionein [3].

Dietary 250 mg/kg Cu significantly impaired feed intake and weight gain in birds fed ZnSO_4_ · H_2_O but had little or no impact in birds fed Zn(HMTBa)_2_. Computer modeling analyses suggest that the interaction between HMTBa and Zn (at a 2:1 molar ratio) is more stable over a range of physiological pH values compared to the interaction between methionine and Zn (at a 1:1 molar ratio) (unpublished observations). In turn, Zn(HMTBa)_2_ would be predicted to be more stable in solution than Zn-MET, and less susceptible to dietary antagonisms over the variable pH range found in the gastrointestinal tract. Thus, the inherent physicochemical features of Zn(HMTBa)_2_ might contribute to greater overall bioavailability and superior performance compared to the ZnSO_4_ · H_2_O evaluated here. Collectively, the data reported here indicate that Zn(HMTBa)_2_ was less susceptible to Cu-mediated antagonism compared to ZnSO_4_ · H_2_O. Furthermore, bioavailability experiments using tissue zinc levels and zinc-responsive gene expression as indicators of bioavailability have shown that Zn(HMTBa)_2_ exhibits greater relative bioavailability than these other sources [11, 17, 18].

In experiments such as these, careful consideration must be given to the response range of the outcome measures (eg weight gain, tissue Zn, etc.). Differences between Zn sources can only be determined at low intakes (below the requirement or inflection point). Homeostatic physiological mechanisms preclude the demonstration of differences between Zn sources, once the supplementation level is above the inflection point [8, 11, 19]. At deficient intake, Zn absorption is maximized. Bioavailability is significantly dependent on the degree of absorption. In purified diets, in the absence of phytate, antagonisms, or a challenge (lipopolysaccharides, etc.), there is little difference in utilization between organic and inorganic sources. In commercial diets or phytate-containing diets, absorption of organic sources of minerals are depressed, but to a lesser extent than inorganic sources. For example, it was demonstrated by Wedekind et al. that the inflection point or breakpoint varies among Zn sources [8]. The breakpoint or inflection point for bone Zn was determined to be 54, 60 and 65 mg total Zn per kg/diet for Zn-MET, ZnSO_4_ and ZnO, respectively for birds fed a corn-SBM diet. Their studies also showed an inflection point for weight gain for ZnSO_4_ for chicks fed a soy isolate diet to occur at 33 mg Zn/kg diet [20]. Comparison of Zn sources above the inflection point would result in an underestimation of the bioavailability or performance difference that may truly exist between Zn sources. These findings were confirmed in our studies. Marked differences between Zn(HMTBa)_2_ and ZnSO_4_ · H_2_O were observed at 30 and 45 mg Zn/kg diet, but not at higher levels. In the presence of excess Cu (250 mg Cu/kg diet), the requirement or inflection point for weight gain, although not defined in this study, is likely higher than the 33 mg Zn/kg determined by Wedekind et al., wherein no excesses of Ca or Cu were present [8].

Our study had some limitations and strengths. A limitation of this study was the amount of dietary Cu selected for the elevated Cu condition. For environmental reasons, the European Union and China restrict the maximum amount of Cu in animal feed. The 250 mg/kg diet CuSO_4_ is above the maximum allowed levels in these geographic areas, so the level used in this study might not be relevant for these regions. However, the mineral antagonism demonstrated in our studies is still important, since antagonism could potentially happen at lower levels of Cu supplementation, and 250 mg/kg dietary Cu is a level used commercially in the United States. Organic trace minerals, in general, and Zn(HMTBa)_2_, specifically, offer an alternative solution to address the challenge of providing required dietary minerals, at lower levels, to meet animal nutritional requirements while avoiding potential antagonism with other nutrients. Furthermore, in Experiment 2, differences between two Zn sources were only observed at 30 and 45 mg/kg, not at 60 and 75 mg/kg. This suggests that the Zn requirement (in the presence of 250 mg/kg Cu) is between 45 and 60 mg/kg. In future studies, dietary Zn supplementation should be chosen below the inflection point, to more sensitively assess bioavailability differences among different Zn sources. Finally, in future studies, biological function parameters should be measured to understand how Cu antagonizes Zn, along with the physiological consequences of that antagonism. For example measurement of metallothionein, collagen, and immune function have served as reliable indices in previous studies. More research is needed to understand the biological consequences of Cu-Zn antagonism in addition to performance and mineral storage in tissues.

The statistical design of our studies and the use of an experimental basal diet containing phytate were some of the strengths of our studies. A factorial design offers advantages over one-way ANOVA studies. There is more power to measure main effects as well as the ability to measure interactions. Significant interactions or trends were observed in all three experiments which demonstrated the improved performance of chelated Zn(HMTBa)_2_ vs ZnSO_4_ · H_2_O in the presence of elevated Cu. The presence of phytate, common in cereals and grains, is an important antagonist that reduces Zn bioavailability. In the presence of antagonisms (ie, phytate, fiber, elevated Cu, Ca, P, etc.), bioavailability differences between OTM and ITM are increased [20]. The presence of elevated Cu and phytate are conditions that are relevant to diets fed commercially to poultry and livestock. The advantages of the Zn(HMTBa)_2_ chelate demonstrated in our study under conditions of elevated Cu has also been demonstrated under other antagonistic conditions (eg, elevated Ca and P) [11]. Bioavailability of Zn(HMTBa)_2_, relative to ZnSO_4_ · H_2_O was 161 % (total bone Zn) and 248 % (metallothionein) in the presence of typical Ca and P (0.82 % Ca and 0.47 % available P). However, in the presence of elevated Ca and P (1.2 % Ca; 1 % available P) bioavailability of Zn(HMTBa)_2_, relative to ZnSO_4_ · H_2_O was even greater: 441 % (total bone Zn) and 426 % (metallothionein) [11].

The use of high dietary CuSO_4_ as a growth promoter is common practice in both the broiler and swine industries in North America and some other geographical areas. The bioavailability of other nutrients (Zn and P) should be considered when high CuSO_4_ is used. One practical strategy is to increase the level of addition of these other nutrients as is often practiced in today’s broiler industry. However, this could result in additional antagonisms along with excess nutrient excretion to the environment. Organic trace minerals have the potential advantage of providing dietary minerals that are more bioavailable. Consequently, less mineral is required to achieve a similar performance level compared to inorganic trace minerals [21, 22]. However, not all OTMs are equally capable of avoiding these antagonisms, and thus do not always provide equivalent bioavailability. More research is needed to fully decipher the mode of action of OTMs and their benefits, compared to inorganic sources of Zn, along with understanding the differences among different OTMs.

## Conclusions

Dietary 250 mg/kg Cu significantly impaired feed intake and weight gain in birds fed ZnSO_4_ · H_2_O and had little or no impact in birds fed Zn(HMTBa)_2_. No significant differences were observed between Zn(HMTBa)_2_ and Zn-Met.

## Abbreviations

MW: molecular weight
OTM(s): organic trace mineral(s)
Zn(HMTBa)_2_: [Zn bis(−2-hydroxy-4-(methylthio)butanoic acid)] at 2:1 ratio
Zn-MET: zinc methionine
